# Long-Term Outcomes of Conservative Versus Invasive Approach of Coronary Aneurysm

**DOI:** 10.31083/j.rcm2308281

**Published:** 2022-08-10

**Authors:** Anthony Matta, Francisco Campelo-Parada, Vanessa Nader, Thibault Lhermusier, Frédéric Bouisset, Stéphanie Blanco, Meyer Elbaz, Jerome Roncalli, Didier Carrié

**Affiliations:** ^1^Department of Cardiology, Toulouse University Hospital (Hopital Rangeuil), 31400 Toulouse, France; ^2^Department of Cardiology, Centre Hospitalier Intercommunal Castres-Mazamet, 81108 Castres, France; ^3^Faculty of Sciences, Paul-Sabatier Toulouse III University, 31062 Toulouse, France; ^4^Faculty of Medicine, Holy Spirit University of Kaslik, 446 Jounieh, Lebanon

**Keywords:** coronary artery aneurysm, medical, percutaneous coronary intervention, cardiac surgery, coronary artery disease

## Abstract

**Introduction:**

Up to date, the management of coronary artery aneurysm (CAA) is not well defined and depends on local heart team decision. Data reported in literature are scarce and controversial. We aim to compare the long-term outcomes of different therapeutic strategies of CAA (medical vs percutaneous coronary intervention (PCI) vs coronary artery bypass graft(CABG)).

**Materials and Methods:**

A retrospective cohort study was conducted on 100 consecutive patients who underwent coronary angiography at Toulouse University Hospital, Toulouse France and fulfilled the diagnostic criteria of CAA. Coronary angiograms were reviewed, and all necessary data were collected. CAA was defined by a coronary dilation exceedingly at least 50% of reference coronary diameter.

**Results:**

We identified 100 patients with CAA with a mean age of 67.9 ± 12 years. The left anterior descending coronary artery was most affected (36%). CAA is associated with significant coronary artery disease in 78% of cases. The incidence of major adverse cardiovascular and cerebrovascular events (MACCE) was 13% during a median follow-up period of 46.2 ± 24 months. A 53% of patients underwent PCI or CABG. The rate of MACCE was lower in CABG group (9.1%) compared to PCI (14.3%) and medical (12.8%) groups, but without reaching statistically significant level. Longitudinal aneurysm diameter was positively linked to MACCE [OR = 1.109, 95% CI (1.014–1.214), *p* = 0.024]. No benefits have been attributed to anticoagulant regimen over antiplatelet therapy.

**Conclusions:**

In our retrospective observational study, there seems to be no significant differences in MACCE-free survival between all groups (Medical vs PCI vs CABG). Larger longitudinal aneurysm diameter was identified as a predictor of poor prognosis during follow-up.

## 1. Introduction

**Fig. 1.**
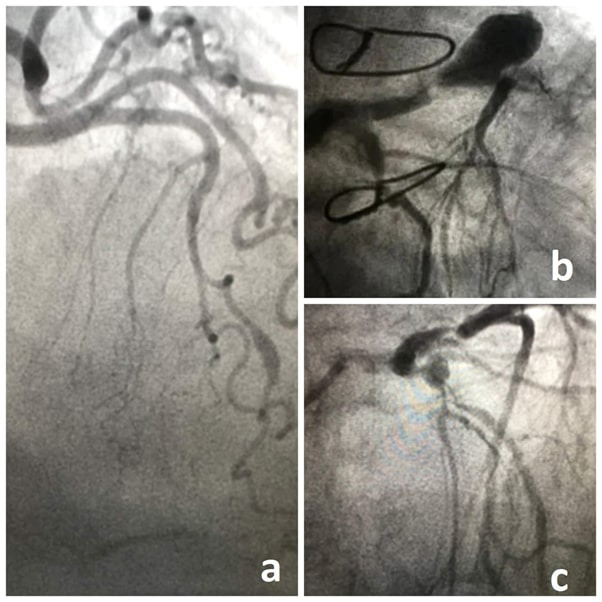
**Coronary angiograms showing fusiform aneurysm of the left anterior descending (LAD) (a), giant saccular aneurysm of the LAD (b) and saccular aneurysm of the bifurcation LAD/diagonal (c)**.

Coronary artery aneurysm (CAA) is defined as an abnormal focal enlargement of the coronary artery exceedingly at least 50% of the reference vessel diameter [1]. The reported incidence of CAA is rare and varies between 0.02 to 5.3% [2, 3, 4] with predilection to men over women and to proximal over distal coronary segments [4]. CAA is mainly divided into 2 types according to the anatomical morphology: fusiform aneurysms when longitudinal diameter is larger than transverse diameter and saccular aneurysms in the adverse case (Fig. 1). An aneurysmal dilation exceeding four folds the diameter of adjacent normal coronary segment forms a giant CAA [5] (Fig. 1). The pathogenesis of CAA is not well understood, and causal agents include atherosclerosis, genetic susceptibility, inflammatory disorders, infectious diseases, connective tissue disorders, trauma, drug reactions and iatrogenic conditions (stent angioplasty, atherectomy) [6, 7, 8]. In general, CAA is a silent disease incidentally detected by coronary angiography; however, it may lead to life-threatening complications such as thrombus formation, distal embolization, coronary steal syndrome, acute rupture and mechanical compression of adjoining structures. To date, there is no agreement on the proper management of CAA. Data from literature are controversial and limited to few small studies, case series and anecdotal evidence. Different therapeutic approaches based on medical treatment, percutaneous coronary intervention and cardiac surgery have been reported [2, 6, 9, 10]. The long-term outcomes of CAA and the prognostic value of each therapy remain unclear, thereby decision to intervene on CAA is individualized depending on its characteristics (form, size, location), clinical implication, technical challenges, coexistence of obstructive coronary artery disease and physician experience. The impact of antithrombotic therapy and the outcome of different available regimens remain unclear and not yet defined [6]. Herein, we conducted a retrospective cohort study comparing the long-term major cardiac and cerebrovascular events (MACCE) of patients with CAA who were treated conservatively versus those who underwent invasive interventions (PCI or surgery) over a 46-months follow-up period.

## 2. Materials and Methods 

### 2.1 Study Design and Population

An observational retrospective cohort study was conducted on patients referred to coronary angiography at Toulouse University Hospital, Toulouse-France between September 2013 and April 2021. We searched in our digital database for all coronary angiography reports including the term coronary aneurysm. Coronary angiograms were reviewed and patients with coronary artery ectasia were excluded from the study. A total of 100 patients (108 CAAs) were included in this study. According to the performed therapeutic approach, the study participants were divided into two groups: the conservative group including those treated medically versus the invasive group including those treated by percutaneous coronary intervention (PCI) or cardiac surgery.

### 2.2 Data Collection and End Points

We searched for medical reports including the term aneurysm in our digital database for cardiac catheterization. Coronary angiography films of the 100 included patients were reviewed by the same physician “AM”. Data concerning the anatomical details of CAA (type, transverse and longitudinal diameters, location), coexistence of significant CAD (≥50% reduction in coronary lumen independently from the CAA position), baseline characteristics, cardiovascular risk factors and medical treatment were collected. Quantitative coronary analysis was used to measure the reference coronary diameter, transverse and longitudinal aneurysm diameters. We also observed for the occurrence of major adverse cardiac and cerebrovascular events (MACCE) defined by any major cardiovascular events (myocardial infarction, cardiac death, coronary revascularization), cerebrovascular events (ischemic or hemorrhagic stroke) and all-causes of death during the period between coronary angiography exam and last available follow-up. The primary end point is to compare MACCE-free survival between the different therapeutic approaches.

### 2.3 Statistical Analysis

Frequency and percentage were calculated for categorical variables while continuous variables were expressed by means and standard deviation. Continuous variables were compared with the use of *t*-test or Mann & Whitney (Medical vs Invasive) or ANOVA (Medical vs PCI vs CABG), as appropriate, and categorical variables with the use of Chi-square test or Fischer’s exact test, as appropriate. Normality tests for continuous variables were performed. Adjusted stepwise logistic regression were conducted to assess the association between the performed therapeutic strategies and MACCE. Kaplan-Meier curves and log Rank test were used for survival analysis. A two-sided *p*-value < 0.05 was considered of statistical significance.

## 3. Results 

The mean age of the study population was 64.9 ± 12 years and 18% of the study participants were women. CAA has been found in 100 over 26,962 coronary angiograms resulting in a prevalence of 0.37%. The indication of coronary angiography referral was acute coronary syndrome (32%), atypical or typical angina with silent ischemia (30%), heart failure (11%) and miscellaneous (pre-surgery, valvular disease workup) (27%). Out of the 100 included patients, 47% were treated conservatively while 53% underwent invasive approaches like percutaneous coronary intervention (42%) and coronary artery bypass graft (CABG) (11%). Percutaneous coronary intervention was markedly more performed in patients with acute coronary syndrome as initial presentation (61.3% vs 33.3%, *p* = 0.012). The proportion of CAA associated with obstructive coronary artery disease (CAD) was 78% and it was significantly more common in the invasive group [100% (CABG), 88.1% (PCI) vs 63.8% (medical)]. Also, multi-vessels coronary disease is more commonly observed in the invasive group (Table 1). The proportion of patients receiving oral anticoagulant is larger in those without versus with CAD (27.3% vs 23.1%). Also, the use of oral anticoagulant was higher in patients with giant CAA compared to the rest of the study population (33.3% vs 21.3%). Among patients without CAD, 17 (77.3%) were treated medically while percutaneous coronary intervention was performed in the remaining 5 patients (22.7%). Except for dyslipidemia and smoking, no significant statistical differences in the prevalence of cardiovascular risk factors (diabetes mellitus, arterial hypertension, chronic kidney disease, familial history and obesity) among the study groups were noted (Table 1). The half of the reviewed CAAs were saccular. The mean transverse and longitudinal diameters of CAA were 7.23 ± 2.2 mm and 10.42 ± 6.39 mm, respectively. Indeed, the mean of aneurysm transverse diameter differs significantly between groups, and it was longer in the CABG subgroup [8.54 ± 2.81 mm vs 6.7 ± 1.9 mm (PCI) vs 7.38 ± 2.15 mm (medical)]. Aneurysmal dilation affects frequently the left anterior descending artery (36%) followed by the right coronary artery (34%), left circumflex artery (24%) and left main (5%). The number of atherosclerotic vessels disease and the distribution of CAA on coronary arteries vary significantly between the study groups. The baseline and demographic characteristics of the study population have been shown in Table 1.

**Table 1.** **Baseline and demographic characteristics of study population with coronary artery aneurysms**.

		Studied population (N = 100)	Conservative approach (N = 47)	Invasive approach (N = 53)		*p*-value
				PCI (N = 42)	CABG (N = 11)	
Age (years)		64.9 ± 12.6	66.1 ± 11.3	64.8 ± 14.2	60 ± 10.7	0.364
BMI (kg/$m^{2}$)		27.4 ± 5.3	27.6 ± 5.8	26.8 ± 4.8	28.9 ± 4.9	0.464
LVEF (%)		52.3 ± 10.4	52.7 ± 12.2	51.4 ± 9.1	54.2 ± 5.8	0.276
Women (n, %)		18 (18)	8 (17)	9 (21.4)	1 (9.1)	0.810
Dyslipidemia (n, %)		52 (52)	20 (42.6)	25 (59.5)	7 (63.6)	0.199
Diabetes mellitus (n, %)		25 (25)	10 (21.3)	10 (23.8)	5 (45.5)	0.256
Hypertension (n, %)		62 (62)	30 (63.8)	24 (57.1)	8 (72.7)	0.611
Smoking (n, %)		28 (28)	9 (19.1)	15 (35.7)	4 (36.4)	0.171
Chronic kidney disease (n, %)		34 (34)	18 (38.3)	12 (28.6)	4 (40)	0.589
Significant CAD (n, %)		78 (78)	30 (63.8)	37 (88.1)	11 (100)	0.003
Single-vessel disease (n, %)		30 (30)	16 (34)	14 (33.3)	0 (0)	<0.05
Two-vessels disease (n, %)		29 (29)	12 (25.5)	14 (33.3)	3 (27.3)	
Three-vessels disease (n, %)		19 (19)	2 (4.3)	9 (21.4)	8 (72.7)	
Type of CAA (n, %)						0.548
	Fusiform	54 (50)	27 (52.9)	22 (48.9)	5 (41.7)	
	Saccular	54 (50)	24 (47.1)	23 (51.1)	7 (58.3)	
Longitudinal aneurysm diameter (mm)		10.4 ± 6.4	10.7 ± 6.6	10.2 ± 6.7	9.7 ± 4	0.276
Transverse aneurysm diameter (mm)		7.2 ± 2.2	7.4 ± 2	6.7 ± 1.9	8.5 ± 2.8	0.039
Giant CAA (n, %)		21 (19.4)	13 (25.5)	4 (8.9)	4 (33.3)	0.042
Involved coronary (n, %)						0.051
	LAD	36 (36)	21 (44.7)	11 (26.8)	4 (36.4)	
	RCA	34 (34)	11 (23.4)	20 (48.8)	3 (27.3)	
	CX	24 (24)	12 (25.2)	10 (24.4)	2 (18.2)	
	LM	5 (5)	3 (6.4)	0 (0)	2 (18.2)	
MACCE (n, %)		13 (13)	6 (12.8)	6 (14.3)	1 (9.1)	1
Follow-up (months)		46.2 ± 24	47.1 ± 24	47.3 ± 23.7	38 ± 25	0.495
Anti-thrombotic treatment (n, %)						0.009
	None	5 (5)	5 (10.6)	0 (0)	0 (0)	
	Aspirin or P2Y12-	40 (40)	21 (44.7)	11 (26.2)	8 (72.7)	
	Dual anti-platelet	30 (30)	10 (21.3)	19 (45.2)	1 (9.1)	
	Oral anticoagulant	25 (25)	11 (23.4)	12 (28.6)	2 (18.2)	

*BMI, body mass index; LVEF, left ventricular ejection fraction; CAD, coronary artery disease; CAA, coronary artery aneurysm; LAD, left anterior descending; RCA, right coronary artery; CX, circumflex artery; LM, left main; MACCE, major adverse cardiac and cerebrovascular events.

The prevalence of MACCE was 13% over a mean follow-up period of 46.2 ± 24 months. MACCE accounted for five deaths (5%), three ischemic strokes (3%), one hemorrhagic stroke (1%) and four coronary revascularizations (4%). No significant differences in MACCE have been observed if CAA was associated or not with CAD (Table 2).

**Table 2.** **Characteristics of the study population stratified by the occurrence of major adverse cardiac and cerebrovascular events (MACCE)**.

		Studied population (N = 100)	MACCE-group (N = 13)	No MACCE-group (N = 87)	*p*-value
Age (years)		64.9 ± 12.6	70.3 ± 17.9	64.1 ± 11.5	0.099
BMI (kg/$m^{2}$)		27.4 ± 5.3	25.8 ± 4.3	27.6 ± 5.4	0.267
LVEF (%)		52.3 ± 10.4	48 ± 10.9	52.9 ± 10.3	0.116
Women (n, %)		18 (18)	4 (30.8)	14 (16.1)	0.243
Dyslipidemia (n, %)		52 (52)	2 (15.4)	45 (51.7)	0.886
Diabetes mellitus (n, %)		25 (25)	2 (15.4)	23 (26.4)	0.508
Hypertension (n, %)		62 (62)	9 (69.2)	53 (60.9)	0.761
Smoking (n, %)		28 (28)	3 (35.7)	25 (28.7)	0.171
Chronic kidney disease (n, %)		34 (34)	7 (53.8)	27 (31.4)	0.128
Acute coronary syndrome as initial presentation (n, %)		32 (32)	5 (38.5)	27 (31)	0.533
Significant CAD (n, %)		78 (78)	11 (84.6)	67 (77)	0.727
Type of CAA (n, %)					0.357
	Fusiform	54 (50)	9 (60)	45 (48.4)	
	Saccular	54 (50)	6 (40)	48 (51.6)	
Longitudinal aneurysm diameter (mm)		10.4 ± 6.4	13.5 ± 8.3	9.98 ± 6	0.074
Transverse aneurysm diameter (mm)		7.2 ± 2.2	7.2 ± 1.3	7.23 ± 2.3	0.974
Giant CAA (n, %)		21 (19.4)	2 (12.5)	19 (20.6)	1
Therapeutic strategy (n, %)					1
	Medical	47 (47)	6 (46.2)	41 (47.1)	
	PCI	42 (42)	6 (46.2)	36 (41.4)	
	CABG	11 (11)	1 (7.7)	10 (11.5)	
Conservative approach (n, %)		47 (47)	6 (46.2)	41 (47.1)	0.948
Invasive approach (n, %)		53 (53)	7 (53.8)	46 (52.9)	
Anti-thrombotic treatment (n, %)					0.170
	None	5 (5)	0 (0)	5 (5.7)	
	Aspirin or P2Y12-	40 (40)	7 (53.8)	33 (38)	
	Dual anti-platelet	30 (30)	1 (7.7)	29 (33.3)	
	Oral anticoagulant	25 (25)	5 (38.5)	20 (23)	

*MACCE, major adverse cardiac and cerebrovascular events; BMI, body mass index; LVEF, left ventricular ejection fraction; CAD, coronary artery disease; CAA, coronary artery aneurysm.

The 13 study participants who develop adverse clinical outcomes or MACCE during the follow up period were older (70.3 ± 17.9 vs 64.1 ± 11.5), frequently smokers (35.7% vs 28.7%) with chronic kidney disease (53.8% vs 31.4%), reduced left ventricular ejection fraction (LVEF) (48 ± 10.9% vs 52.9 ± 10.2%) and longer mean longitudinal aneurysm dimension (13.5 ± 8.2 mm vs 9.9 ± 6 mm) (Table 2). The multivariate logistic regression identified the longitudinal aneurysm diameter as independent predictor of MACCE [OR = 1.109, 95% CI (1.014–1.214), *p* = 0.024] and revealed a negative relationship between LVEF and MACCE [OR = 0.935, 95% CI (0.880–0.994), *p* = 0.032] (Table 3). However, the distribution of the prevalence of MACCE between the therapeutic groups (Medical, PCI and CABG) was not statistically significant (12.8% vs 14.3% vs 9.1%) (Table 1). In parallel, the rate of different therapeutic approach modalities (conservative or invasive) was almost similar between those who developed MACCE overtime versus others (46.2% vs 47.1% for conservative management, 53.8% vs 52.9% for invasive management) (Table 2).

**Table 3.** **Multivariate logistic regression identifying the independent predictors of major adverse cardiovascular and cerebrovascular events [The results were similar if treatment was introduced as categorical variable (Medical vs PCI vs CABG) or as dichotomic variable (Invasive vs Conservative)]**.

		OR	95% CI	*p*-value
Age		1.033	[0.970–1.101]	0.313
Longitudinal diameter		1.109	[1.014–1.214]	0.024
LVEF		0.935	[0.880–0.994]	0.032
Chronic kidney disease		2.958	[0.616–14.211]	0.176
Smoking		1.656	[0.312–8.791]	0.554
Treatment				0.787
	PCI	1.642	[0.402–6.719]	0.490
	CABG	1.410	[0.120–16.515]	0.784

*LVEF, left ventricular ejection fraction; PCI, percutaneous coronary intervention; CABG, coronary artery bypass graft.

**Fig. 2.**
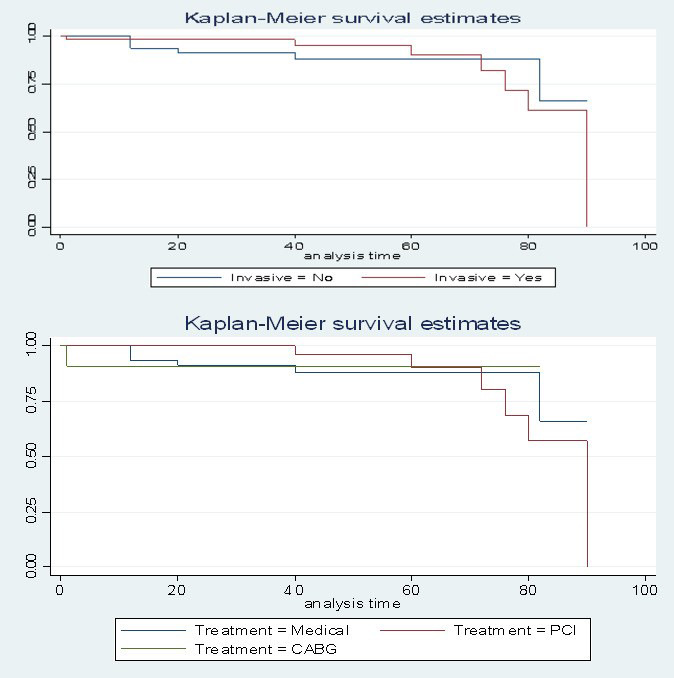
**Kaplan-Meier survival analysis for freedom of major adverse cardiovascular and cerebrovascular events (MACCE) in patients with invasive versus conservative treatment and in patients with medical management versus PCI versus CABG**.

**Fig. 3.**
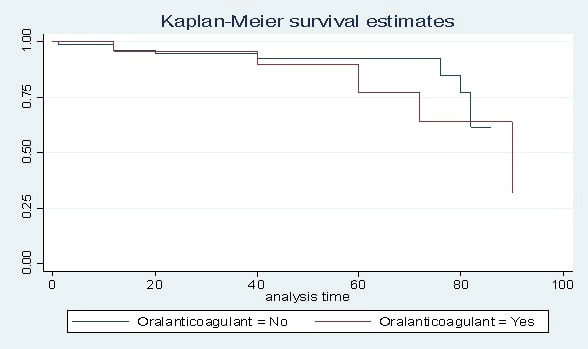
**Kaplan-Meier survival analysis for freedom of major adverse cardiovascular and cerebrovascular events (MACCE) in patients who received oral anticoagulant versus others**.

Lastly, the Kaplan-Meier Curve and log-Rank test failed to detect a significant difference in survival between the study groups [Conservative vs Invasive, *p* = 0.821 (Fig. 2)] and subgroups [Medical vs PCI vs CABG, *p* = 0.957 (Fig. 2)], respectively. Pointing on the antithrombotic regimen, 40% received single antiplatelet, 30% dual antiplatelet and 25% oral anticoagulant without significant different distribution between study participants who developed MACCE and others (MACCE- vs No MACCE – groups) (Table 2). As expected, dual antiplatelet treatment was more frequent among PCI-subgroup (Table 1). There was no significant difference for MACCE free survival in favor of those receiving oral anticoagulant, *p* = 0.557 (Fig. 3).

## 4. Discussion

The results of this study showed an absence of significant difference in MACCE free survival at 46 months follow up between the different proposed treatment strategies in patients with CAA. However, the longitudinal aneurysm diameter was positively associated to MACCE. The presence of obstructive CAD and coexistence of multi-vessels coronary disease were in favor of invasive approach. The prevalence of CAA was 0.37% of the total catheterization reports and it is close to the previously reported ones in literature that range between 0.2% to 5.3% [11, 12].

In line with recently published studies [9, 11, 12], LAD was the most common involved coronary artery and atherosclerosis was predominantly associated to CAA. They share multiple histological features such as focal calcification, lipid deposition, fibrosis and alteration of vascular layers [13, 14]. In fact, atherosclerosis is a chronic inflammatory disease affecting the transmural vascular wall from the tunica intima to the external elastic lamina, thereby modifying the architecture of coronary artery walls in a manner to decrease the resistance to intraluminal pressure ending with progressive dilation and aneurysm formation [13, 14, 15, 16]. Up to date, the exact pathophysiological mechanism of CAA is not well known, but atherosclerosis is the main reported cause in adults and Kawasaki disease in children [2]. Also, vasculitis, proteolytic imbalance, genetic susceptibility, infectious diseases and iatrogenic conditions (post-percutaneous coronary intervention) may contribute to CAA formation. The coexistence of CAD plays a pivotal role in the choice of therapeutic strategy [6] and constitutes the main determinant parameter for invasive approach as shown by this study result. Herein, 100% of patients treated with CABG (N = 11) have diffuse CAD (27.2% two- and 72.7% three vessels disease). Noteworthy that these interventions were not exclusively performed for the treatment of CAA, but also for the associated obstructive CAD. Studies comparing the outcomes of invasive versus conservative management of CAA are scarce in literature. In our study, no significant differences between conservative and invasive (PCI+CABG) strategies and between medical, PCI and CABG subgroups have been found. In parallel, data from the International Coronary Artery Aneurysm Registry (CAAR) reported similar rates of mortality and MACCE among those treated by CABG or PCI. A similar finding was revealed by a smaller study conducted on 42 patients who underwent CABG (18/42) and PCI (24/42) for CAA [17]. The study by Khubber *et al*. [11], including 230, 176 and 52 participants in medical, CABG and PCI groups, respectively has attributed a better outcome to CABG over medical treatment but like that of PCI. In our study, the prevalence of MACCE was lower in CABG group (9.1%) than that in PCI (14.3%) and medical (12.8%) groups, but without reaching the level of statistical significance. The overall low prevalence of MACCE (13%) in the studied population and the small number of patients who underwent cardiac surgery (11%) may limit the power of this study to detect a statistical difference in favor of CABG. According to previously published data, larger aneurysm size and coexisting of heart failure are predictors of poor prognosis [11, 18, 19]. Indeed, we showed a positive association between the occurrence of adverse clinical outcomes, longitudinal aneurysm diameter and reduced LVEF. Lastly, no benefits for anticoagulant regimen over single or dual antiplatelet therapy have been observed. Results from previously published studies concerning antithrombotic therapy in CAA patients are conflicting. Thereby, some studies describe a similar finding to this study result while others conclude for positive effects of anticoagulation [6]. In fact, anti-thrombotic regimens were influenced by the therapeutic strategy and most probably by other co-morbidities. Thus, dual antiplatelet therapy was largely overexpressed in the PCI-subgroup, and this makes sense. Among patients treated with anticoagulants, 7% had atrial fibrillation and 3% had thromboembolic event as a reason for oral anticoagulant.

## 5. Limitations 

The observational retrospective study design that may predispose to selection bias and immortal time bias. The low prevalence of adverse clinical outcomes limits the power of the study to determine the potential predictive factors of MACCE. Also, the small sample size makes difficult to clearly conclude on the impact of anti-thrombotic therapy. In parallel, the number of study participants in CABG group is low which subsequently reduces the risk to detect a statistical difference in favor of this approach. Invasive versus conservative therapy was chosen based on the Heart Team’s clinical judgment in the best interest of the patient at the time of the procedure. We can suppose that most severe patients with coexisting CAD were treated by CABG or PCI.

## 6. Conclusions

CAA is frequently associated with CAD. Patients with multi-vessels disease are more predisposed to undergo invasive therapeutic approach (PCI and CABG). Data analysis of long-term MACCE free survival showed similar outcomes in medical, PCI and CABG groups. Longitudinal aneurysm diameter was positively associated with MACCE. Also, no additional benefits have been observed with oral anti-coagulant regimen. However, this conclusion must be carefully interpreted in view of the limitation of retrospective study design and the small sample size, thereby larger randomized prospective multi-centric trials are required to better understand and optimize the management of CAA.
